# Response to letter regarding: “sarcopenia is a bad harbinger of cancer-related survival in rectal cancer”

**DOI:** 10.1080/07853890.2026.2635854

**Published:** 2026-02-24

**Authors:** Sema Yilmaz Rakici

**Affiliations:** Department of Radiation Oncology, Recep Tayyip Erdogan University Faculty of Medicine, Rize, Türkiye

Dear Editor

We would like to thank the authors for their thoughtful and constructive letter regarding our study entitled *“Sarcopenia is a Bad Harbinger of Cancer-Related Survival in Rectal Cancer”* [1,2]. We appreciate their balanced appraisal and the opportunity to clarify several methodological and clinical points.

As highlighted, a key strength of our work is its clinical feasibility. By using routinely acquired radiotherapy planning CT images within the treatment planning system, quantitative body composition parameters can be obtained without additional imaging, dedicated software, or extra radiation exposure. The excellent inter-observer agreement further supports the reproducibility and scalability of this approach in daily radiotherapy practice.

We agree that our measurements are derived from a single axial CT slice at the L3 level and should therefore be interpreted as density-segmented cross-sectional tissue volumes, rather than true three-dimensional volumetric assessments. Our intention was not to replace established definitions of sarcopenia, but rather to propose a practical, workflow-integrated surrogate capable of identifying patients at increased risk using data already available during radiotherapy planning. The modest discrimination observed in the ROC analyses reflects this pragmatic aim and should be interpreted as a triage signal rather than a definitive diagnostic threshold.

We acknowledge that reporting height-adjusted skeletal muscle index values and incorporating functional measures such as handgrip strength or gait speed would improve alignment with contemporary sarcopenia frameworks. However, the retrospective nature of our study and the lack of routinely collected functional data precluded such analyses. This limitation underscores the need for prospective studies integrating radiologic, biochemical, and functional assessments.

Regarding the biochemical findings, we agree that the prognostic prominence of post-treatment albumin levels warrants careful interpretation. Hypoalbuminemia following neoadjuvant therapy likely reflects a complex interplay of nutritional depletion, systemic inflammation, treatment-related toxicity, and hepatic dysfunction. Its emergence as the sole independent predictor in multivariable analysis suggests that albumin may function both as a global risk marker and as a potentially modifiable intermediate endpoint, supporting future evaluation of nutritional and prehabilitation interventions during neoadjuvant treatment.

Finally, we concur that prospective, multicenter studies with predefined sarcopenia criteria, serial measurements, and stratification by operability and metastatic status are required to validate and extend our findings. Indeed, recent evidence indicates that sarcopenia has prognostic significance across multiple cancer types [3,4]. From this perspective, incorporating a practical method for sarcopenia assessment into radiotherapy planning may facilitate its effective integration into routine oncology practice.

We thank the authors once again for their valuable comments and for contributing to a constructive discussion on the clinical integration of sarcopenia assessment in rectal cancer.

## Data Availability

Data sharing is not applicable to this article as no data were created or analysed in this research.
